# Nutritional-inflammatory status and diabetic retinopathy: exploring the association between advanced lung cancer inflammation index and retinal complications in diabetes

**DOI:** 10.3389/fnut.2025.1602361

**Published:** 2025-06-30

**Authors:** Jinxiang Peng, Zhuang Chen, Yanqiong Wang, Kui Wang, Feng Wu, Jianjun Xiang

**Affiliations:** ^1^School of Chinese Medicine, Hubei University of Chinese Medicine, Wuhan, China; ^2^Medical Department, Hubei Enshi College, Enshi, Hubei, China; ^3^Fifth People’s Hospital of Jinan, Jinan, Shangdong, China; ^4^Enshi Central Hospital, Enshi, Hubei, China; ^5^Department of Pulmonary Disease Diabetes Mellitus, The Central Hospital of Enshi Tujia and Miao Autonomous Prefecture, Enshi, Hubei, China

**Keywords:** advanced lung cancer inflammation index, diabetic retinopathy, inflammation, nutrition, metabolic health, NHANES, non-linear association

## Abstract

**Objective:**

This study aimed to assess the association between the advanced lung cancer inflammation index (ALI) and the prevalence of diabetic retinopathy (DR) in a nationally representative sample of US adults with diabetes.

**Methods:**

We used cross-sectional data from the National Health and Nutrition Examination Survey (NHANES) spanning 1999 to 2018. ALI was calculated from the body mass index (BMI), albumin levels, and neutrophil-to-lymphocyte ratio (NLR), providing an integrative measure of inflammatory and nutritional metabolic status. A history of diabetes was obtained through self-report. Logistic regression models were used to investigate the relationship between ALI and DR prevalence, adjusting for multiple potential confounders. Additionally, restricted cubic spline (RCS) analyses were used to explore potential non-linear associations.

**Results:**

A total of 3,952 diabetic participants were included, of whom 813 had DR. Logistic regression analysis shows that higher ALI values are significantly correlated with a decrease in DR prevalence. Compared to the lowest ALI quartile, the highest quartile was associated with a 27% decrease in DR prevalence after full adjustment. Subgroup analyses showed that the relationship remained stable across most demographic and clinical strata, although racial differences were also observed. Furthermore, RCS analyses revealed an L-shaped relationship between ALI and DR prevalence.

**Conclusion:**

In the US adult diabetic population, lower ALI levels were associated with greater DR prevalence, and this relationship displayed an L-shaped, non-linear pattern. These findings suggest that monitoring and managing ALI may be beneficial in reducing the risk of DR. Future longitudinal studies are needed to clarify the causality and evaluate the impact of ALI-targeted interventions in clinical practice.

## 1 Introduction

Diabetic retinopathy (DR) is one of the most prevalent and severe microvascular complications of diabetes, characterized by pathological alterations in the retinal vasculature triggered by persistent hyperglycemia. This condition leads to endothelial damage, increased vascular permeability, microhemorrhage, and neovascularization, potentially resulting in visual impairment (1, 2). Current treatments for DR include laser photocoagulation, intravitreal injections of anti-VEGF agents, and surgical procedures, such as vitrectomy (3). However, the multifactorial nature of DR, which involves hyperglycemia-induced metabolic disturbances, low-grade inflammation, and complex lipid dysregulation, poses significant challenges. Therefore, developing robust strategies for prevention, early detection, and timely intervention remains imperative (4–6).

Globally, approximately the 30–40% of individuals with diabetes develop DR, creating substantial clinical, economic, and psychological burden. With diabetes prevalence rising worldwide, identifying accessible and integrative biomarkers that reflect underlying metabolic and inflammatory processes is crucial for improving risk stratification and guiding targeted interventions (7–9).

In recent years, the advanced lung cancer inflammation index (ALI), originally introduced as a prognostic indicator in patients with advanced lung cancer, has gained attention in various diseases. ALI integrates metrics such as body mass index, albumin levels, and neutrophil-to-lymphocyte ratio, combining nutritional status and systemic inflammatory burden into a single measure. Although initially established in oncology, research suggests that ALI may have predictive value in metabolic and vascular conditions (10–12). However, its role in diabetic microvascular complications, including DR, remains underexplored.

Drawing on data from the National Health and Nutrition Examination Survey (NHANES) from 1999 to 2018, the present cross-sectional study investigated the association between ALI and DR prevalence among US adults with diabetes. This is the first comprehensive effort to evaluate this integrated inflammation-nutrition index in the context of DR risk. Confirming this relationship could facilitate new approaches for risk profiling and interventions tailored to the inflammatory and nutritional dimensions of DR pathogenesis (13). To our knowledge, this is the first large-scale epidemiological study to investigate the relationship between ALI and DR in a nationally representative sample. By repurposing ALI—originally developed for oncology—as a potential biomarker for diabetic microvascular complications, we introduce a novel perspective that integrates inflammatory and nutritional dimensions into DR risk assessment. The findings of this study will not only offer novel insights into the systemic nature of DR pathogenesis but also guide future longitudinal and interventional research aimed at improving clinical outcomes for individuals with diabetes through targeted anti-inflammatory and nutritional interventions.

## 2 Materials and methods

### 2.1 Source of data and study population

The data for this study were obtained from the National Health and Nutrition Examination Survey (NHANES), a long-standing program initiated in the late 1960s to assess the health and nutritional status of the US population. The NHANES employs a complex multistage probability sampling design to ensure national representativeness. Data are released biennially, and encompass a comprehensive array of demographic, health, nutritional, and biochemical variables. The most recent available dataset extends to 2018, encompassing a wealth of health and nutrition-related information. The NHANES aims to monitor and evaluate the health of US residents, providing critical scientific evidence to support public health policies and clinical practices (14).

**FIGURE 1:**
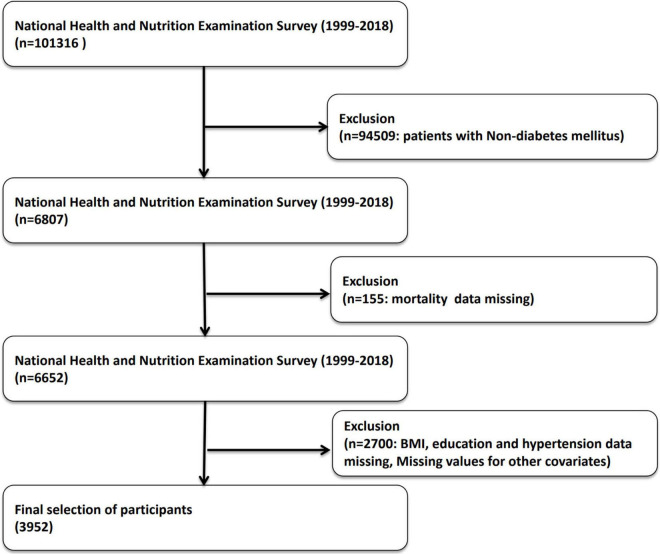
Flowchart of participant inclusion and exclusion.

This study utilized a cross-sectional design, focusing on participants with diabetes in the NHANES from 1999 to 2018. NHANES data are collected continuously and released every two years, providing nationally representative information. The initial study population included 101,316 individuals. Participants were excluded based on the following criteria: (1) absence of diabetes data and (2) missing covariate data. After applying these exclusion criteria, 3,952 diabetic patients were included in the analysis (see Figure 1). This study evaluated the cross-sectional relationship between advanced lung cancer inflammation index (ALI) and prevalence of diabetic retinopathy (DR).

### 2.2 Definition of ALI

ALI served as the primary exposure variable in this study and was calculated using the following formula: ALI = BMI × Albumin/NLR, where BMI is body mass index (kg/m^2^), Albumin is serum albumin level (g/dL), and NLR is neutrophil-to-lymphocyte ratio. ALI reflects an individual’s systemic inflammatory status and nutritional profile, offering a composite metric with a demonstrated predictive value in various diseases. Its emerging role as a biomarker of inflammation and lipid metabolism highlights its relevance in microvascular complications such as DR (15).

### 2.3 Assessment of DM and diabetic visual impairment

Diabetes was defined as having a fasting plasma glucose (FPG) level of more than 126 mg/dL or a glycated hemoglobin (HbA1c) level of at least 6.5% or having a physician-diagnosed diagnosis of DM. Diabetic retinopathy (DR) was identified through the NHANES standardized questionnaire protocol, which includes validated health condition assessment questions administered by trained interviewers. Participants were classified as having DR based on their response to the specific question “Has diabetes affected your eyes/have you had retinopathy?” This methodology aligns with NHANES-recommended practices for population health surveillance and epidemiological research. The use of this assessment approach enables comprehensive analysis across multiple survey cycles (1999–2018), facilitating robust statistical power and nationally representative estimates that would be challenging to achieve with clinical examinations in such a large-scale population study.

### 2.4 Measurement of covariates

Multiple potential confounders were incorporated as covariates, including age, sex, race, education level, income level (Poverty Income Ratio, PIR), smoking and alcohol use, history of angina, myocardial infarction, heart failure, hypertension, high-density lipoprotein (HDL) level, and total cholesterol level. Specific measurement protocols for these covariates are detailed on the NHANES website (NHANES, CDC).

### 2.5 Statistical analysis

All analyses were performed using SAS (version 9.4) and R (version 4.0.3) software. Continuous variables are expressed as medians with interquartile ranges, while categorical variables are represented as percentages. The Chi-square test was used to assess differences in categorical variables, and the Mann-Whitney U test was used to analyze continuous variables. Logistic regression models were used to explore the relationship between ALI and DR prevalence, with DR as the binary dependent variable. The study tested ALI both as a continuous variable and categorized into quartiles: Model 1: Unadjusted. Model 2: Adjusted for age, sex, race, education, smoking, and alcohol consumption. Model 3: Further adjusted for history of angina, myocardial infarction, heart failure, and hypertension. Model 4: Additionally adjusted for HDL and total cholesterol levels.

Restricted cubic spline (RCS) fitting were used to investigate the potential non-linear relationships between ALI and DR prevalence. Stratified analyses and interaction tests were conducted across subgroups defined by age, sex, race, education, income, smoking, alcohol consumption, and comorbidities including cardiovascular history. Statistical significance was set at *P* < 0.05.

## 3 Results

### 3.1 Baseline characteristics of the study population

A total of 3,952 patients with diabetes, comprising 2,291 males and 1,661 females. Among all participants, 813 had diabetic retinopathy (DR), while 3,139 did not. Table 1 shows that participants in the higher quartiles of ALI generally exhibited lower age and higher BMI values. Significant differences (all *P* < 0.05) were observed among the different ALI quartiles in terms of race, education level, smoking status, history of angina, myocardial infarction, heart failure, and hypertension. Specifically, higher ALI quartiles were associated with higher BMI and lower age. Additionally, the high ALI group had a higher proportion of African Americans, lower education levels, and a higher prevalence of smoking.

**TABLE 1:** Demographics and characteristics of study participants from NHANES 1999–2018.

		Advanced lung cancer inflammation index				
Variables	Total (*n* = 3,952)	Quartile 1 (*n* = 988)	Quartile 2 (*n* = 988)	Quartile 3 (*n* = 988)	Quartile 4 (*n* = 988)	*P*-value
**Gender, *n* (%)**						< 0.001
Male	2,291 (58.0)	675 (68.3)	566 (57.3)	560 (56.7)	490 (49.6)	
Female	1,661 (42.0)	313 (31.7)	422 (42.7)	428 (43.3)	498 (50.4)	
Age (years)	61.2 ± 13.0	65.2 ± 12.8	62.2 ± 13.2	59.1 ± 12.6	58.3 ± 12.3	< 0.001
**Race, *n* (%)**						< 0.001
Mexican American	733 (18.5)	164 (16.6)	212 (21.5)	200 (20.2)	157 (15.9)	
Other Hispanic	350 (8.9)	90 (9.1)	82 (8.3)	105 (10.6)	73 (7.4)	
Non-Hispanic White	1,532 (38.8)	476 (48.2)	417 (42.2)	356 (36)	283 (28.6)	
Non-Hispanic Black	1,028 (26.0)	165 (16.7)	212 (21.5)	241 (24.4)	410 (41.5)	
Other race	309 (7.8)	93 (9.4)	65 (6.6)	86 (8.7)	65 (6.6)	
**Educational level, *n* (%)**						0.006
Less than 9th grade	634 (16.0)	150 (15.2)	171 (17.3)	156 (15.8)	157 (15.9)	
9–11th grade	670 (17.0)	169 (17.1)	165 (16.7)	151 (15.3)	185 (18.7)	
High school grad/GED or equivalent	891 (22.5)	235 (23.8)	218 (22.1)	224 (22.7)	214 (21.7)	
Some college or AA degree	1,135 (28.7)	250 (25.3)	294 (29.8)	283 (28.6)	308 (31.2)	
College graduate or above	622 (15.7)	184 (18.6)	140 (14.2)	174 (17.6)	124 (12.6)	
**Smoking, *n* (%)**						< 0.001
Yes	2,275 (57.6)	616 (62.3)	588 (59.5)	520 (52.6)	551 (55.8)	
No	1,677 (42.4)	372 (37.7)	400 (40.5)	468 (47.4)	437 (44.2)	
**Alcohol use, *n* (%)**						0.279
Yes	853 (21.6)	222 (22.5)	204 (20.6)	198 (20)	229 (23.2)	
No	3,099 (78.4)	766 (77.5)	784 (79.4)	790 (80)	759 (76.8)	
**Heart failure, *n* (%)**						< 0.001
Yes	407 (10.3)	142 (14.4)	107 (10.8)	82 (8.3)	76 (7.7)	
No	3,545 (89.7)	846 (85.6)	881 (89.2)	906 (91.7)	912 (92.3)	
**Angina pectoris, *n* (%)**						0.004
Yes	314 (7.9)	102 (10.3)	78 (7.9)	59 (6)	75 (7.6)	
No	3,638 (92.1)	886 (89.7)	910 (92.1)	929 (94)	913 (92.4)	
**Heart attack, *n* (%)**						< 0.001
Yes	476 (12.0)	166 (16.8)	114 (11.5)	102 (10.3)	94 (9.5)	
No	3,476 (88.0)	822 (83.2)	874 (88.5)	886 (89.7)	894 (90.5)	
**Hypertension, *n* (%)**						0.005
Yes	2,719 (68.8)	656 (66.4)	669 (67.7)	670 (67.8)	724 (73.3)	
No	1,233 (31.2)	332 (33.6)	319 (32.3)	318 (32.2)	264 (26.7)	
Total cholesterol	4.7 ± 1.2	4.5 ± 1.2	4.7 ± 1.1	4.8 ± 1.2	4.9 ± 1.2	< 0.001
HDL	1.2 ± 0.4	1.3 ± 0.4	1.2 ± 0.4	1.2 ± 0.4	1.3 ± 0.4	0.303
Albumin	41.1 ± 3.5	40.3 ± 4.0	41.1 ± 3.3	41.5 ± 3.3	41.6 ± 3.3	< 0.001
Lymphocyte	2.2 ± 1.2	1.5 ± 0.5	2.0 ± 0.5	2.3 ± 0.7	2.9 ± 2.0	< 0.001
Neutrophil	4.6 ± 1.7	5.6 ± 1.9	4.9 ± 1.5	4.3 ± 1.4	3.5 ± 1.3	< 0.001
BMI	32.4 ± 7.4	29.0 ± 5.7	31.6 ± 6.6	33.5 ± 7.2	35.5 ± 8.4	< 0.001
**DR, *n* (%)**						0.002
Yes	813 (20.6)	244 (24.7)	181 (18.3)	196 (19.8)	192 (19.4)	
No	3,139 (79.4)	744 (75.3)	807 (81.7)	792 (80.2)	796 (80.6)	

DR, diabetic retinopathy; BMI, body mass index; HDL, high density lipoprotein.

### 3.2 Association between ALI and prevalence of diabetic retinopathy

Our analysis indicated that higher levels of ALI were significantly associated with an decreased prevalence of DR. In the unadjusted model, compared to participants in the lowest quartile of ALI, those in the second, third, and fourth quartiles had odds ratios (OR) for DR of 0.68 (95% CI: 0.55–0.85), 0.75 (95% CI: 0.61–0.93), and 0.74 (95% CI: 0.59–0.91). After adjusting for age, sex, race, education level, smoking, and alcohol consumption in Model 2, the ORs were 0.7 (95% CI: 0.56–0.87), 0.77 (95% CI: 0.62–0.96), and 0.73 (95% CI: 0.58–0.92) for the second, third, and fourth quartiles, respectively, remaining statistically significant (Table 2). These results suggest that ALI, as a composite indicator, is inversely associated with DR prevalence, with higher ALI values corresponding to lower odds of DR.

**TABLE 2:** Association between advanced lung cancer inflammation index and the prevalence of diabetic retinopathy.

ALI	OR (95% CI)							
	Model 1	*P*-value	Model 2	*P*-value	Model 3	*P*-value	Model 4	*P*-value
Quartile 1	1.00 (reference)		1.00 (reference)		1.00 (reference)		1.00 (reference)	
Quartile 2	0.68 (0.55∼0.85)	0.001	0.69 (0.55∼0.86)	0.001	0.7 (0.56∼0.87)	0.001	0.7 (0.56∼0.87)	0.001
Quartile 3	0.75 (0.61∼0.93)	0.01	0.76 (0.61∼0.94)	0.012	0.77 (0.62∼0.96)	0.019	0.77 (0.62∼0.96)	0.023
Quartile 4	0.74 (0.59∼0.91)	0.005	0.72 (0.57∼0.9)	0.004	0.72 (0.58∼0.91)	0.006	0.73 (0.58∼0.92)	0.007
*P*-trend	0.013		0.011		0.016		0.02	

Model 1, crude model; Model 2, adjusted for gender, age, race, educational level, smoking and alcohol use; Model 3, adjusted for gender, age, race, educational level, smoking, alcohol use, heart failure, angina pectoris, heart attack, hypertension; Model 4, adjusted for gender, age, race, educational level, smoking, alcohol use, heart failure, angina pectoris, heart attack, hypertension, total cholesterol and high- density lipoprotein. OR, Odds ratio; CI, confidence interval.

### 3.3 Results of restricted cubic spline analysis

**FIGURE 2:**
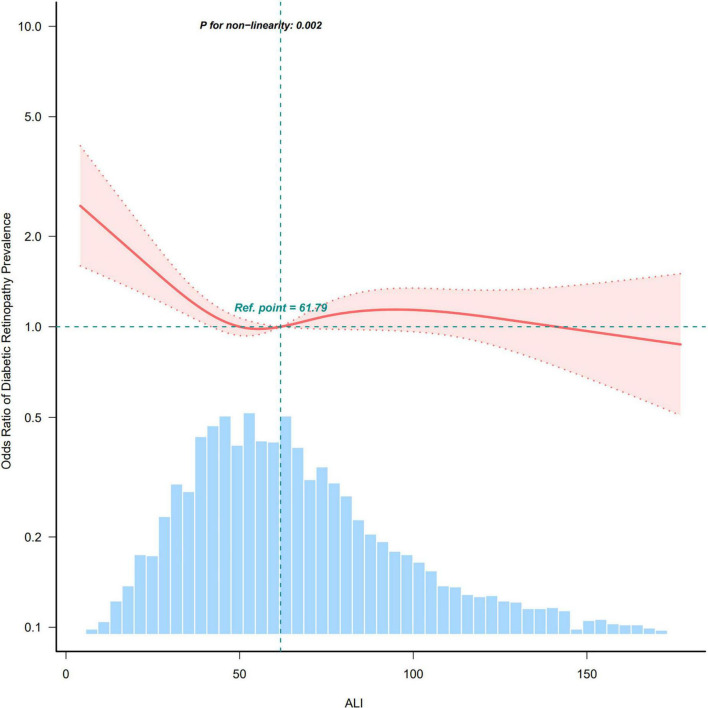
Restricted cubic spline curve illustrating the relationship between ALI and DR prevalence.

After adjusting for all covariates, we utilized restricted cubic spline (RCS) analysis to explore the relationship between ALI and the prevalence of DR. The results demonstrated a significant non-linear relationship between the two variables (Figure 2). Specifically, the ALI and DR prevalence exhibited an L-shaped curve, indicating that the protective effect of ALI against DR is most pronounced at lower ALI values and tends to plateau at higher values. Specifically, DR risk decreases sharply as ALI increases from the lowest values until approximately the median, after which additional increases in ALI confer diminishing protective benefits. From a clinical perspective, this relationship holds significant importance. This finding suggests that ALI may serve as a simple clinical tool for identifying high-risk DR patients, particularly for those individuals with notably low ALI values.

### 3.4 Subgroup analysis

To verify the stability of the association between ALI and DR prevalence across different populations, multifactorial subgroup analyses were conducted (Table 3). The analyses included subgroups based on sex, age, race, education level, income level (PIR), smoking, alcohol consumption, history of myocardial infarction, history of heart failure, history of angina, hypertension, and diabetes. The results indicated that, except for race, the association between ALI and DR remained consistent across all subgroups, suggesting that the negative association between ALI and DR has high generalizability and stability. The protective association between higher ALI and lower DR prevalence was significantly stronger in African–American populations compared to other racial groups (*P* < 0.05). This suggests that African Americans may particularly benefit from interventions targeting ALI components, though the mechanisms underlying this racial difference require further investigation.

**TABLE 3:** The results of subgroup analysis.

Subgroup	Variable	OR (95% CI)	*P*-value	*P* for trend	*P* for interaction
**Gender**					0.925
Male				0.098	
	Quartile 1	1 (reference)			
	Quartile 2	0.68 (0.51∼0.89)	0.005		
	Quartile 3	0.79 (0.61∼1.04)	0.089		
	Quartile 4	0.77 (0.58∼1.02)	0.069		
Female				0.12	
	Quartile 1	1 (reference)			
	Quartile 2	0.71 (0.49∼1.02)	0.062		
	Quartile 3	0.72 (0.5∼1.03)	0.072		
	Quartile 4	0.72 (0.51∼1.02)	0.065		
**Age**					0.802
< 60 years				0.243	
	Quartile 1	1 (reference)			
	Quartile 2	0.6 (0.4∼0.89)	0.011		
	Quartile 3	0.76 (0.53∼1.09)	0.131		
	Quartile 4	0.71 (0.49∼1.02)	0.061		
≥ 60 years					
	Quartile 1	1 (reference)		0.033	
	Quartile 2	0.73 (0.56∼0.95)	0.017		
	Quartile 3	0.74 (0.57∼0.98)	0.032		
	Quartile 4	0.75 (0.57∼0.99)	0.04		
**Race**					0.153
Mexican American	Quartile 1	1 (reference)		0.322	
	Quartile 2	0.8 (0.48∼1.33)	0.393		
	Quartile 3	0.97 (0.59∼1.61)	0.917		
	Quartile 4	0.67 (0.38∼1.19)	0.17		
Other Hispanic				0.166	
	Quartile 1	1 (reference)			
	Quartile 2	0.28 (0.13∼0.61)	0.001		
	Quartile 3	0.57 (0.3∼1.06)	0.074		
	Quartile 4	0.55 (0.28∼1.1)	0.091		
Non-Hispanic White				0.358	
	Quartile 1	1 (reference)			
	Quartile 2	0.79 (0.57∼1.1)	0.168		
	Quartile 3	0.7 (0.49∼1)	0.05		
	Quartile 4	0.92 (0.64∼1.33)	0.674		
Non-Hispanic Black				0.149	
	Quartile 1	1 (reference)			
	Quartile 2	0.89 (0.55∼1.44)	0.636		
	Quartile 3	0.88 (0.55∼1.41)	0.596		
	Quartile 4	0.73 (0.48∼1.13)	0.162		
Other race				0.053	
	Quartile 1	1 (reference)			
	Quartile 2	0.32 (0.14∼0.7)	0.004		
	Quartile 3	0.46 (0.24∼0.9)	0.023		
	Quartile 4	0.52 (0.25∼1.06)	0.073		
**Educational level**					0.091
Less than 9th grade				0.133	
	Quartile 1	1 (reference)			
	Quartile 2	0.58 (0.34∼0.97)	0.037		
	Quartile 3	0.86 (0.52∼1.42)	0.552		
	Quartile 4	0.57 (0.33∼0.97)	0.037		
9–11th grade					
	Quartile 1	1 (reference)		0.932	
	Quartile 2	1.03 (0.61∼1.75)	0.91		
	Quartile 3	0.99 (0.57∼1.7)	0.968		
	Quartile 4	0.99 (0.59∼1.66)	0.969		
High School Grad/GED or Equivalent				0.035	
	Quartile 1	1 (reference)			
	Quartile 2	1 (0.64∼1.55)	0.991		
	Quartile 3	0.71 (0.45∼1.12)	0.139		
	Quartile 4	0.66 (0.41∼1.05)	0.08		
Some College or AA degree				0.662	
	Quartile 1	1 (reference)			
	Quartile 2	0.51 (0.34∼0.79)	0.002		
	Quartile 3	0.82 (0.55∼1.21)	0.314		
	Quartile 4	0.78 (0.52∼1.15)	0.206		
College Graduate or above				0.061	
	Quartile 1	1 (reference)			
	Quartile 2	0.44 (0.24∼0.8)	0.007		
	Quartile 3	0.43 (0.25∼0.76)	0.003		
	Quartile 4	0.68 (0.39∼1.2)	0.184		
**Smoking**					0.202
Yes				0.266	
	Quartile 1	1 (reference)			
	Quartile 2	0.7 (0.53∼0.93)	0.013		
	Quartile 3	0.92 (0.69∼1.21)	0.536		
	Quartile 4	0.79 (0.59∼1.04)	0.093		
No				0.013	
	Quartile 1	1 (reference)			
	Quartile 2	0.65 (0.46∼0.92)	0.015		
	Quartile 3	0.58 (0.42∼0.82)	0.002		
	Quartile 4	0.66 (0.48∼0.93)	0.016		
**Alcohol use**					0.763
Yes				0.278	
	Quartile 1	1 (reference)			
	Quartile 2	0.8 (0.51∼1.25)	0.32		
	Quartile 3	0.9 (0.58∼1.41)	0.646		
	Quartile 4	0.75 (0.48∼1.16)	0.195		
No				0.025	
	Quartile 1	1 (reference)			
	Quartile 2	0.66 (0.51∼0.84)	0.001		
	Quartile 3	0.72 (0.56∼0.92)	0.008		
	Quartile 4	0.73 (0.57∼0.93)	0.012		

ALI, advanced lung cancer inflammation index; OR, Odds ratio; CI, confidence interval.

## 4 Discussion

This study utilized a nationally representative sample of American adults with diabetes to explore the relationship between the advanced lung cancer inflammation index (ALI) and the prevalence of diabetic retinopathy (DR). ALI integrates body mass index (BMI), albumin levels, and neutrophil-to-lymphocyte ratio (NLR), thus reflecting systemic inflammatory, nutritional, and metabolic states. Our analysis revealed a significant L-shaped, non-linear association between ALI and DR prevalence. These observations suggest an association between systemic inflammatory-nutritional states and retinal microvascular status. Furthermore, racial differences moderated this association, underscoring the interplay between genetic, cultural, and socioeconomic factors in DR pathogenesis. These findings expand the applicability of ALI beyond oncology, highlighting its utility in systemic diseases and underscoring the need for a holistic approach for DR risk assessment (4, 16).

Reframing DR within the broader context of systemic health, this study challenges the traditional focus on hyperglycemia-induced microvascular damage (17). While chronic hyperglycemia disrupts endothelial function, induces oxidative stress, and increases vascular permeability (18, 19), our findings suggest that inflammatory and nutritional imbalances are equally pivotal. Additionally, glycoxidation of protein by reactive carbonyl compounds like methylglyoxal has been shown to disturb structural integrity and increase immunogenicity of important serum proteins like IgG, thereby contributing to inflammation and immune dysregulation in T2DM (20). The L-shaped association we observed underscores this complexity: lower ALI values may represent inadequate nutrition and increased inflammation, impairing repair mechanisms of retinal vasculature (21), while higher ALI values likely signify a more favorable metabolic environment with better nutritional status and lower inflammatory burden (22, 23). These observations align with emerging research on the role of inflammation in DR pathogenesis, where pro-inflammatory cytokines like TNF-α and IL-1β exacerbate vascular leakage and pathological angiogenesis (7). A comparison with existing literature further corroborates the clinical relevance of ALI (24). Previous studies have linked ALI to outcomes in coronary heart disease, chronic kidney disease, and diabetic nephropathy, suggesting shared pathophysiological pathways rooted in systemic inflammation and metabolic dysregulation. By demonstrating the association between ALI and DR, our study broadens its clinical scope, establishing it as a systemic health indicator rather than an organ-specific biomarker. Leveraging NHANES data ensures external validity and reduces selection bias, while our advanced statistical methods enable rigorous examination of non-linearities and effect modifiers. These methodological strengths underscore our findings: ALI appears to correlate with DR risk based on underlying systemic health. This perspective aligns with integrated care paradigms that emphasize the interplay between systemic and localized disease processes (25–27).

The potential mechanisms linking ALI to diabetic retinopathy are multifaceted. Chronic hyperglycemia triggers a cascade of deleterious changes in the retina, but the three components of ALI collectively reflect additional dimensions of systemic health that may influence retinal microvascular integrity. BMI correlates with metabolic states—high BMI indicates insulin resistance (28), while low BMI may signal malnutrition; albumin reflects nutritional and inflammatory status—low albumin implies chronic inflammation and catabolic stress (29); and NLR serves as a marker of systemic inflammation—elevated NLR indicates neutrophil predominance and lymphocyte suppression (30). By combining these factors, ALI transcends isolated biomarkers to provide a comprehensive measure of the patient’s physiological milieu (31). Poor nutritional status, as reflected by low albumin levels, compromises tissue repair mechanisms and antioxidant defenses, rendering the retina more vulnerable to metabolic insults (11). Furthermore, an abnormal body mass index, whether too low or excessively high, modulates insulin sensitivity, lipid profiles, and adipokine secretion, potentially amplifying microvascular damage (32) The neutrophil-to-lymphocyte ratio emphasizes the chronic inflammatory milieu that may accelerate these processes through increased oxidative stress, endothelial dysfunction, and vascular permeability. This systemic environment likely influences the delicate balance between damage and repair in retinal tissues, with lower ALI reflecting conditions that favor microvascular deterioration. Research suggests that inflammatory cells infiltrate the retina early in DR, where pro-inflammatory cytokines exacerbate vascular leakage and promote neovascularization (9). Thus, ALI emerges as a sentinel that reflects the systemic environment from which DR evolves. Rather than focusing exclusively on advanced retinal changes, ALI may potentially serve as an early indicator of systemic disturbances that precede clinically detectable retinopathy, though longitudinal studies are needed to confirm this temporal relationship.

The potential clinical implications of incorporating ALI into research on diabetes management warrant consideration, though our cross-sectional findings cannot establish causality. Current DR screening protocols primarily focus on glycemic control, diabetes duration, and periodic retinal examination. Evaluating whether complementing these approaches with ALI measurements provides clinically meaningful insights into patients’ systemic environment would require prospective clinical trials. If such studies confirm our findings, several potential applications might emerge. Patients with diabetes who exhibit lower ALI levels might benefit from more frequent retinal screening, even in the absence of other risk factors. Targeted nutritional interventions to improve serum albumin levels and anti-inflammatory strategies to reduce NLR could represent novel approaches to DR prevention, though these require prospective evaluation (33). In resource-limited settings where advanced imaging is inaccessible, ALI’s simplicity and cost-effectiveness could theoretically make it a practical complementary tool for risk stratification, though implementation would require validation studies. As healthcare paradigms shift toward precision medicine and prevention-focused strategies, integrative biomarkers like ALI align with these trends. Beyond individual care, the adoption of ALI has potential broader implications for public health research addressing diabetes complications. Replication of our findings across diverse populations could encourage further investigation into nutritional and inflammatory pathways in DR pathogenesis. On a population level, ALI distributions could identify at-risk subgroups, potentially guiding targeted research into community initiatives such as culturally sensitive dietary programs and exercise campaigns (26, 34–36). This research direction, focusing on upstream factors rather than downstream complications, may ultimately contribute to improved patient outcomes and more efficient use of healthcare resources. However, we emphasize that these potential applications require confirmation through longitudinal and interventional studies before any clinical implementation can be recommended.

Before concluding, it is important to acknowledge several limitations of this study. The cross-sectional design precludes definitive conclusions about causality, as the temporal relationship between ALI elevation and DR development remains undetermined—whether high ALI predisposes individuals to DR or if early DR alters systemic parameters reflected in ALI components. Prospective longitudinal studies are needed to establish these temporal sequences and evaluate if ALI modification reduces DR risk. A notable limitation is our use of self-reported DR data rather than clinical verification, potentially underestimating DR prevalence, especially in milder cases. While pragmatic for large-scale surveys like NHANES, incorporating retinal imaging or standardized ophthalmoscopy would substantially enhance diagnostic accuracy in future studies. The absence of comprehensive glycemic control assessment, particularly HbA1c measurements, represents a significant methodological limitation that introduces interpretative complexity to our findings. Additionally, while ALI integrates key inflammatory and metabolic indicators, its current formulation omits critical variables including glycemic control, blood pressure measurements, and angiogenic factors such as VEGF. Refining ALI or combining it with complementary markers could significantly improve its clinical utility (37). Important unmeasured confounders—genetic factors, dietary patterns, and psychosocial stressors—likely contribute to unexplained variability despite our adjustment efforts. Furthermore, our study did not differentiate between non-proliferative and proliferative DR stages. Future research should explore whether the relationship between ALI and DR varies by retinopathy severity, which could facilitate more targeted preventive strategies. The study’s strengths lie in its large, diverse NHANES sample, enhancing external validity. However, the relatively modest number of DR cases limits statistical power for subgroup analyses and may obscure important interactions. Future pooled analyses across multiple datasets could provide more precise population-specific ALI thresholds and clarify their role in DR pathophysiology (38).

In conclusion, this cross-sectional study identifies a significant inverse association between ALI and DR prevalence, suggesting potential utility of ALI as a risk stratification tool. While our findings suggest ALI could be relevant to DR pathophysiology, prospective studies are necessary before recommending its integration into routine clinical practice.

## Data Availability

The original contributions presented in this study are included in this article/supplementary material, further inquiries can be directed to the corresponding authors.
